# Prognostic study of cardiac events in Japanese patients with chronic kidney disease using ECG-gated myocardial Perfusion imaging: Final 3-year report of the J-ACCESS 3 study

**DOI:** 10.1007/s12350-017-0880-5

**Published:** 2017-04-24

**Authors:** Satoko Nakamura, Yuhei Kawano, Kenichi Nakajima, Hiroki Hase, Nobuhiko Joki, Tsuguru Hatta, Shigeyuki Nishimura, Masao Moroi, Susumu Nakagawa, Tokuo Kasai, Hideo Kusuoka, Yasuchika Takeishi, Mitsuru Momose, Kazuya Takehana, Mamoru Nanasato, Syunichi Yoda, Hidetaka Nishina, Naoya Matsumoto, Tsunehiko Nishimura

**Affiliations:** 1 Division of Hypertension and Nephrology, National Cerebral and Cardiovascular Center, Suita, Japan; 2 Department of Nuclear Medicine, Kanazawa University Hospital, Kanazawa, Japan; 3 Department of Nephrology, Toho University Ohashi Medical Center, Tokyo, Japan; 4 Division of Nephrology, Department of Medicine, Ohmihachiman Community Medical Center, Ohmihachiman, Japan; 5 Saitama Medical University International Medical Center, Hidaka, Japan; 6 Department of Cardiology, Toho University Ohashi Medical Center, Tokyo, Japan; 7 Department of Cardiology, Saiseikai Central Hospital, Tokyo, Japan; 8 Department of Cardiology, Jikei Medical University Aoto Hospital, Tokyo, Japan; 9 National Hospital Organization Osaka National Hospital, Osaka, Japan; 10 Department of Medicine, Fukushima Medical University, Fukushima, Japan; 11 Department of Radiology, Tokyo Women’s Medical University, Tokyo, Japan; 12 Department of Cardiology, Kansai Medical University, Hirakata, Japan; 13 Department of Cardiology, Nagoya Daini Red-Cross Hospital, Nagoya, Japan; 14 Department of Cardiology, Nihon University Itabashi Hospital, Tokyo, Japan; 15 Department of Cardiology, Tsukuba Medical Center Hospital, Tsukuba, Japan; 16 Department of Cardiology, Suruga-dai Nihon University Hospital, Tokyo, Japan; 17 Department of Radiology, Graduate School of Medical Science, Kyoto Prefectural University of Medicine, 465 Kajiicho, Kawara-machi Hirokoji, Kamigyo-ku, 602-8566 Kyoto, Japan

**Keywords:** Cardiovascular morbidity, C-reactive protein, estimated glomerular filtration rate, prognosis

## Abstract

**Background:**

Myocardial perfusion imaging (MPI) is considered useful for risk stratification among patients with chronic kidney disease (CKD), without renal deterioration by contrast media.

**Methods and Results:**

The Japanese Assessment of Cardiac Events and Survival Study by Quantitative Gated SPECT (J-ACCESS 3) is a multicenter, prospective cohort study investigating the ability of MPI to predict cardiac events in 529 CKD patients without a definitive coronary artery disease. All patients were assessed by stress and rest MPI with ^99m^Tc-tetrofosmin and data were analyzed using a defect scoring method and QGS software. Major cardiac events were analyzed for 3 years after registration. The mean eGFR was 29.0 ± 12.8 (mL/minute/1.73 m^2^). The mean summed stress/rest/difference (SSS, SRS, SDS) scores were 1.9 ± 3.8, 1.1 ± 3.0, and 0.8 ± 1.8, respectively. A total of 60 cardiac events (three cardiac deaths, six sudden deaths, five nonfatal myocardial infarctions, 46 hospitalization cases for heart failure) occurred. The event-free survival rate was lower among patients with kidney dysfunction, higher SSS, and higher CRP values. Multivariate Cox regression analysis independently associated SSS ≥8, eGFR <15 (mL/minute/1.73 m^2^), and CRP ≥0.3 (mg/dL) with cardiac events.

**Conclusions:**

Together with eGFR and CRP, MPI can predict cardiac events in patients with CKD.

## Introduction

Chronic kidney disease (CKD) is considered a worldwide public health problem with adverse outcomes.^1^-^4^ Several studies have shown that the adjusted hazard ratio for death and cardiovascular events increases inversely with estimated glomerular filtration rates (eGFR).^1^,^2^

An estimated 13% of the Japanese adult population has CKD.^5^,^6^ Several Japanese studies have indicated that the hazard ratio of the onset of myocardial infarction (MI) is 2.5-fold higher among Japanese men with CKD and without previous cardiovascular diseases (CVD) than in those without CKD^7^,^8^ and in a Japanese population with eGFR <50 mL/minute/1.73 m^2^.^8^

Although coronary angiography (CAG) has been the gold standard for detecting coronary artery disease (CAD), contrast media-induced nephropathy or cholesterol microembolization is serious adverse effects.^9^ Therefore, stress myocardial perfusion imaging (MPI) might be an appropriate method of detecting CAD without renal deterioration among patients with CKD. Our previous and other studies suggested that MPI could predict the outcomes of patients with suspected or extant CAD and those with CKD.^10^-^13^ However, the study by Hakeem et al. is limited because it proceeded at a single center and by the nature of the study population.^13^

Therefore, we started a multicenter prospective cohort study called J-ACCESS 3, to determine the ability of MPI to diagnose CAD and predict the outcomes of patients with CKD who are not definitively diagnosed with CAD. The study aimed to clarify whether MPI can predict cardiac events and to determine whether renal dysfunction combined with MPI abnormalities provides additional prognostic information to conventional markers.

## Materials and Methods

### Study Design and Participants

The design of the multicenter prospective cohort Japanese Assessment of Cardiac Events and Survival Study by Quantitative Gated SPECT (J-ACCESS 3) study has been described elsewhere.^14^,^15^ A total of 549 patients who registered at 62 institutions between April 2009 and September 2010 were assessed by stress-rest MPI within 2 months of registration and information about them including background, treatment, and clinical and imaging findings was collected.

The inclusion criteria comprised age, ≥20 years, scheduled to undergo stress-rest ECG-gated MPI, having suspected ischemic CAD, eGFR, <50 mL/minute/1.73 m^2^, and having at least one of the seven risk factors for CAD or ischemic heart disease, namely hypertension, diabetes mellitus, dyslipidemia, peripheral vascular diseases, currently smoking, family history of juvenile CAD, and history of ischemic stroke.^14^ CKD was diagnosed based on the Japanese equation:^5^

$$ {\text{eGFR }}\left( {{\text{mL}}/{\text{minute}}/ 1. 7 3 {\text{m}}^{ 2} } \right) = 1 9 4 { } \times {\text{ serum creatinine }}\left( {\text{Cr}} \right) \, \left( {{\text{mg}}/{\text{dL}}} \right)^{ - 1.0 9 4} \, \times {\text{ age }}\left( {\text{years}} \right)^{ - 0. 2 8 7} \, \times \, 0. 7 3 9 { }\left( {\text{for women}} \right). $$

The exclusion criteria comprised hemodialysis or peritoneal dialysis, severe valvular heart disease requiring surgery, hypertrophic or dilated cardiomyopathy, prior diagnosis of angina pectoris or MI, and a history of revascularization-percutaneous coronary intervention (PCI) or coronary artery bypass grafting, a history of CAG or multi-detector computed tomography within two months before enrollment, severe arrhythmia affecting ECG-gating, and confirmed bronchospastic pulmonary disease.

**Figure 1:**
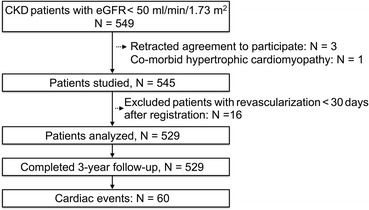
Study design of J-ACCESS 3 and patient registry

Figure 1 shows the findings of an analysis of 529 of 549 patients after 20 of them were excluded due to revascularization within 30 days after MPI (*n* = 16), retracted agreement to participate (*n* = 3), and co-morbid hypertrophic cardiomyopathy (*n* = 1).

The Institutional Review Boards of all participating hospitals approved the study, which proceeded in compliance with the Ethical Guidelines for Epidemiological Research in Japan. All patients provided written informed consent to participate in the study before enrollment.

### Protocol for MPI

One-day (96%) and two-day (4%) pharmacological stress MPI studies proceeded using ^99m^Tc-tetrofosmin, adenosine (91%), dipyridamole (1%), adenosine-triphosphate (6%), and pharmacological agents combined with low-intensity exercise (2%). The average administered dose of ^99m^Tc-tetrofosmin for the first and second studies were 312 and 689 MBq, respectively, and gated MPI was started at 35 ± 17 and 59 ± 45 minute, respectively, after injection. The ECG-gating/cardiac cycle was 16 (65%), 8 (27%), and others (8%). Left ventricular ejection fraction (LVEF), end-diastolic volume (LVEDV), end-systolic volume (LVESV), and other data were quantified using QGS software (Cedars-Sinai Medical Center, Los Angeles, CA, USA).^10^-^12^

### Evaluation of MPI Findings

Tomographic slices were generated using the standard processing protocol verified in previous J-ACCESS studies.^10^-^12^ All single-photon emission-computed tomography (SPECT) data in the Digital Images and Communication in Medicine format were processed at the J-ACCESS office (Osaka, Japan). An image interpretation committee objectively evaluated defect scores in a blinded manner using summed stress/rest/difference scores (SSS/SRS/SDS) with a 17-segment model.^16^ Thresholds for scoring were based on the normal database of the Japanese Society of Nuclear Medicine working group.^17^ The committee visually confirmed computer-generated defect scores ranging from 0 (normal uptake) to 4 (absent uptake).

### Follow-up

The endpoint of this study after 3 years of follow-up comprised the major cardiac events of cardiac death, sudden death, nonfatal MI, and hospitalization due to heart failure (HF).^14^ Cardiac death was defined as death due to HF, MI, and other cardiac disorders. Sudden death was defined when the cause remained unknown within 24 h of occurrence.

### Statistical Analysis

Data are expressed as mean ± SD. Categorical data and mean values between two groups were compared using χ^2^ and Wilcoxon rank sum tests. The three-year follow-up was completed for all patients. In this manuscript, we analyzed the first cardiac events. If a patient experienced several cardiac events during the follow-up, only the first event was counted and analyzed, and thereafter the patients were censored. Differences in cardiac event rates among groups with various SSS, eGFR, and CRP values were compared using Kaplan-Meier estimates. Predictors of cardiac events were assessed using univariate and multivariate Cox proportional hazards analysis. Values with *P* < .05 were considered significant. Goodness of fit was examined using χ^2^ and combination of significant variables. Receiver operating characteristic (ROC) analysis was performed to evaluate differences in models, and area under the curve (AUC) was compared. Net reclassification improvement (NRI) analysis was applied with logistic models for the major cardiac events to evaluate an additional value of CRP. Mortality risk was divided into low and high risk (<10% and ≥10%/3 years).^18^ All data were statistically analyzed using SAS 9.1.3 Service Pack 2 and JMP 12.2 (Cary, NC, USA).

## Results

### Characteristics of the Patients

Table 1 shows the baseline characteristics of the 529 patients with or without cardiac events. The mean age was 71.6 ± 10.9 years, and mean values for Cr and eGFR were 2.2 ± 1.3 mg/dL and 29.0 ± 12.8 mL/minute/1.73 m^2^, respectively. This study population was similar to that of a cohort study of Japanese patients with CKD.^6^ Traditional risk factors in the present study such as smoking, hypertension, diabetes, and dyslipidemia were evident in 6%, 91%, 42%, and 49% of the patients, respectively. Out of 529 patients enrolled in this study, 180 patients (34%) had angina or typical chest pain, 244 (46%) had new onset of dyspnea or palpitation suggestive of ischemic heart disease, and 262 (50%) had ECG abnormalities (positive ischemia on stress ECG, abnormal Q wave, ST segment changes, or T wave changes on rest ECG).

**Table 1:** Characteristics of patients with or without major cardiac events

	Total	Cardiac events		
	N = 529	Yes *n* *=* 60	No *n* *=* 469	*P*
Age (years)	71.6 ± 10.9 (28–92)	71.9 ± 12.7 (33–92)	71.5 ± 10.6 (28–92)	.46
Male (%)	67.3	65.0	67.6	.80
BMI, (kg/m^2^)	24.1 ± 4.1 (14.2–45.9)	24.0 ± 5.0 (16.4–45.9)	24.2 ± 4.0 (14.2–41.6)	.17
Smoking (%)	6	12	6	.14
Hypertension (%)	91	90	91	.94
Diabetes (%)	42	47	41	.48
Dyslipidemia (%)	49	50	48	.92
PAD (%)	8	8	8	1.00
Cerebral infarction (%)	9	7	9	.69
Family history of juvenile CAD (%)	1	2	1	1.00
Typical chest pain (%)	34	35	34	.98
Dyspnea or palpitation suggesting ischemia (%)	46	43	46	.75
ECG abnormalities (%)	50	65	48	.02
SSS	1.9 ± 3.8 (0–29)	3.6 ± 6.3 (0–25)	1.6 ± 3.2 (0–29)	.16
SSS ≥4 (%)	19	30	17	.024
SSS ≥8 (%)	7	18	6	.0010
SRS	1.1 ± 3.0 (0–27)	2.6 ± 5.6 (0–25)	0.9 ± 2.4 (0–27)	.030
SDS	0.8 ± 1.8 (0–12)	1.0 ± 2.3 (0–10)	0.8 ± 1.8 (0–12)	.87
LVEF (%)	61.7 ± 15.0 (17–96)	53.8 ± 14.7 (20–86)	62.7 ± 14.7 (17–96)	<.0001
LVEF <35 (%)	5	10	5	.17
LVEF <40 (%)	9	17	8	.048
LVEDV (mL)	91 ± 39 (18–249)	109 ± 43 (31–224)	88 ± 38 (18–249)	.0001
LVESV (mL)	39 ± 31 (3–184)	54 ± 34 (4–179)	37 ± 30 (3–184)	.0005
Hemoglobin, g/dL	11.7 ± 2.1 (6.6–19.3)	11.0 ± 2.0 (6.6–15.3)	11.8 ± 2.1 (6.7–19.3)	.014
Creatinine (mg/dL)	2.2 ± 1.3 (0.76–10.9)	2.4 ± 1.3 (0.86–6.7)	2.2 ± 1.3 (0.76–10.9)	.035
eGFR (mL/minute/1.73 m^2^)	29.0 ± 12.8 (3.0–71.3)	25.6 ± 13.2 (5.8–53.1)	29.4 ± 12.7 (3.0–71.3)	.032
eGFR <30 mL/minute/1.73 m^2^ (%)	52	63	51	.092
eGFR <15 mL/minute/1.73 m^2^ (%)	19	28	17	.065
Triglyceride (mg/dL)	154 ± 97 (10-954)	152 ± 88 (37–401)	155 ± 98 (10–954)	.80
LDL-C (mg/dL)	108 ± 40 (8-492)	106 ± 39 (9–221)	108 ± 40 (8–492)	.91
HDL-C (mg/dL)	48 ± 15 (21-117)	47 ± 18 (26–117)	48 ± 14 (21–111)	.20
CRP (mg/dL)	0.4 ± 1.0 (0-10.1)	0.8 ± 1.6 (0–6.9)	0.4 ± 0.9 (0–10.1)	.002
CRP ≥0.3 mg/dL (%)	26	47	24	<.0003
HbA_1c_ (%)	6.0 ± 1.1 (4.0-15.5)	6.1 ± 1.2 (4.5–9.6)	5.9 ± 1.1 (4.0–15.5)	.78
ACE-I (%)	14	20	13	.27
ARB (%)	64	60	65	.21
Statin (%)	42	43	41	1.00
Beta blocker (%)	30	38	29	.36
Aspirin (%)	30	32	29	1.00

*ACE-I*, angiotensin-converting inhibitor; *ARB*, angiotensin receptor blocker; *BMI*, body mass index; *CRP*, C-reactive protein; *LVEF*, left ventricular ejection fraction; *eGFR*, glomerular filtration rate; *HbA*_*1c*_, hemoglobin A_1c_; *HDL-C*, high-density lipoprotein cholesterol; *LDL-C*, low-density lipoprotein cholesterol; *PAD*, peripheral artery disease; *SDS*, summed difference score; *SRS*, summed rest score; *SSS*, summed stress score

### MPI Findings

The mean SSS, SRS, and SDS were 1.9 ± 3.8, 1.1 ± 3.0, and 0.8 ± 1.8, respectively (Table 1). Stress myocardial perfusion abnormalities defined as SSS ≥8 were identified in 7% of the patients. The mean LVEF was 61.7 ± 15.0%, and 5% of the patients had reduced cardiac function with LVEF <35%.

### Cardiac Events

Sixty major cardiac events that occurred during the three-year follow-up included cardiac death (*n* *=* 3), sudden death (*n* *=* 6), nonfatal MI (*n* *=* 5), and hospitalization due to HF (*n* *=* 46). Out of 529 patients, the fatal events of cardiac death and sudden death were observed in 9 (1.7%), and hard events including death and nonfatal MI in 14 (2.6%). To identify major cardiac events excluding severe heart failure (*n* *=* 14), univariate proportional hazard analysis showed that SSS ≥8 was the only significant variable (χ^2^ = 4.8, *P* = 0.028 by Wald test). We therefore analyzed major cardiac events including HF hospitalization (*n* *=* 60).

The traditional risk factors of age, sex, smoking, hypertension, diabetes, and dyslipidemia did not significantly differ between patients with and without cardiac events (Table 1). Among nontraditional risk factors, concentrations of Cr and CRP were higher and those of eGFR were lower, in patients with, than without cardiac events. Patients with cardiac events were more likely to have SSS ≥4 or ≥8, LVEF lower, and higher LVEDV and LVESV than those without such events.

### Survival Curves

**Figure 2:**
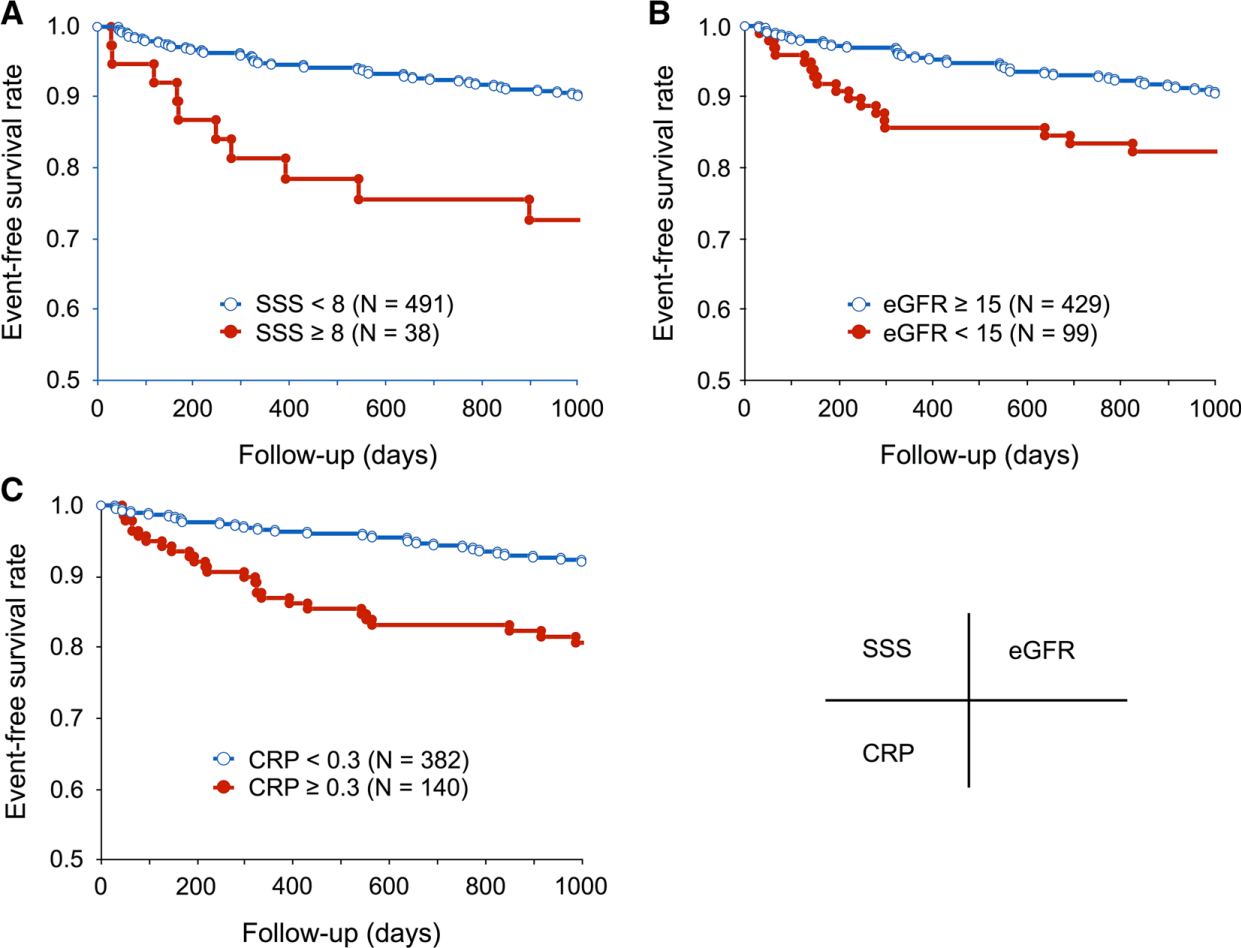
Kaplan-Meier curves of event-free survival rates according to stress MPI findings from patients with CKD. **A** Major event rates significantly differed between patients with SSS <8 and ≥8 (*P* < .001). **B** Major event rates significantly differ between patients with eGFR <15 (mL/minute/1.73 m^2^) and ≥15 (mL/minute/1.73 m^2^) (*P* < .026). **C** Major event rates significantly differ between patients with CRP <0.3 (mg/dL) and ≥0.3 (mg/dL) (*P* < .001)

**Figure 3:**
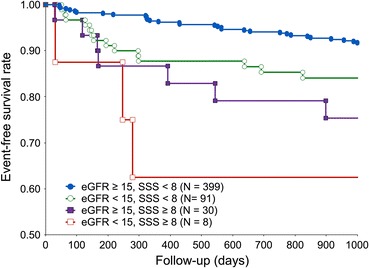
Kaplan-Meier curves of event-free survival rates according to combined SSS and eGFR among patients with CKD. Rates significantly differ among four groups of patients (*P* < .001)

Figures 2 and 3 show Kaplan-Meier curves for event-free survival during 3 years of follow-up. Event-free survival rates significantly differed between groups with SSS <8 and ≥8 (*P* < .001; Figure 2A), eGFR <15 mL/minute/1.73 m^2^ and ≥15 mL/minute/1.73 m^2^ (*P* < .026; Figure 2B), and CRP <0.3 mg/dL and ≥0.3 mg/dL (*P* < .001; Figure 2C). Event-free rates significantly differed among patients assigned to four groups according to combined SSS and eGFR (*P* < .001; Figure 3).

### Potential Predictors for Cardiac Events

Significant predictors for cardiac events were analyzed using univariate Cox proportional hazard analysis (Table 2). Lower eGFR (mL/minute/1.73 m^2^), larger LVEDV and LVESV, lower LVEF and higher CRP values, SSS and SRS were significantly associated with cardiac events.

**Table 2:** Hazard ratios based on univariate Cox proportional hazards analysis to predict major cardiac events

	Wald χ^2^	Hazard Ratio	Lower 95%	Upper 95%	*P*
Age, per year	0.14	1.005	0.981	1.029	.71
Male vs female	0.22	1.135	0.667	1.928	.64
BMI (kg/m^2^)	0.11	0.989	0.926	1.056	.74
Smoking vs nonsmoking	3.11	2.033	0.924	4.471	.08
Hypertension	0.11	0.868	0.374	2.018	.74
Diabetes	0.96	1.289	0.776	2.140	.33
Dyslipidemia	0.02	1.039	0.627	1.724	.88
Peripheral artery disease	0.26	1.268	0.507	3.169	.61
Cerebral infarction	0.37	0.728	0.264	2.008	.54
Typical chest pain	0.007	1.023	0.602	1.738	.93
Dyspnea or palpitation suggesting ischemia	0.17	0.899	0.539	1.498	.68
ECG abnormalities	6.33	1.976	1.163	3.359	.012
SSS, per increment	16.55	1.090	1.046	1.136	<.0001
SRS, per increment	19.77	1.103	1.056	1.151	<.0001
SDS, per increment	0.82	1.057	0.937	1.192	.37
LVEF, per %	19.35	0.967	0.952	0.981	<.0001
LVEDV, per mL	16.61	1.011	1.006	1.016	<.0001
LVESV, per mL	17.09	1.012	1.006	1.018	<.0001
Hemoglobin, per g/dL	7.85	0.822	0.716	0.943	.005
Creatinine, per mg/dL	3.41	1.166	0.991	1.373	.065
eGFR, per mL/minute/1.73m^2^	5.46	0.976	0.956	0.996	.020
Triglyceride, per mg/dl	0.10	1.000	0.997	1.002	.75
LDL-C, per mg/dl	0.23	0.998	0.992	1.005	.63
HDL-C, per mg/dl	0.25	0.995	0.978	1.014	.62
CRP, per mg/dL	10.78	1.258	1.097	1.443	.001
HbA1c, per %	0.86	1.096	0.903	1.329	.35
ACE-I, user vs nonuser	1.65	1.514	0.804	2.850	.20
ARB, user vs nonuser	1.96	0.691	0.412	1.159	.16
Statin, user vs nonuser	0.01	0.974	0.585	1.623	.92
Beta blocker, user vs nonuser	1.13	1.326	0.788	2.231	.29
Aspirin, user vs nonuser	0.001	1.010	0.586	1.740	.97

*ACE-I*, angiotensin-converting inhibitor; *ARB*, angiotensin receptor blocker; *BMI*, body mass index; *CRP*, C-reactive protein; *LVEF*, left ventricular ejection fraction; *eGFR*, glomerular filtration rate; *HbA1*_*c*_, hemoglobin A1_c_; *HDL-C*, high-density lipoprotein cholesterol; *LDL-C*, low-density lipoprotein cholesterol; *PAD*, peripheral artery disease; *SDS*, summed difference score; *SRS*, summed rest score; *SSS*, summed stress score

To identify the independent variables of cardiac events, significant variables (*P* < .05) by univariate analysis were included in a multivariate Cox proportional hazards analysis. The multivariate analysis based on categorical variables showed that eGFR <15 mL/minute/1.73 m^2^, CRP ≥0.3 mg/dL, and SSS ≥8 were significant variables to predict major cardiac events (Table 3).

**Table 3:** Multivariate Cox proportional hazards analysis to identify independent factors associated with onset of major cardiac events

Categorical variables	Univariate				Multivariate			
	Hazard ratio	95% CI		*P*	Hazard ratio	95% CI		*P*
		Lower	Upper			Lower	Upper	
SSS ≥8	3.366	1.750	6.476	<.001	2.68	1.36	5.31	.005
eGFR <15 mL/minute/1.73 m^2^	1.870	1.066	3.297	.030	1.79	1.02	3.16	.042
CRP ≥0.3 mg/dL	2.718	1.630	4.531	<.001	2.51	1.50	4.20	<.001
SSS ≥4	1.992	1.147	3.460	.015				
LVEF <40	2.260	1.145	4.463	.019				
eGFR <30 mL/minute/1.73 m^2^	1.709	1.011	2.890	.046				

*CI*, confidence interval; *CRP*, C-reactive protein; *eGFR*, estimated glomerular filtration rate; *SSS*, summed stress score; *LVEF*, left ventricular ejection fraction

**Figure 4:**
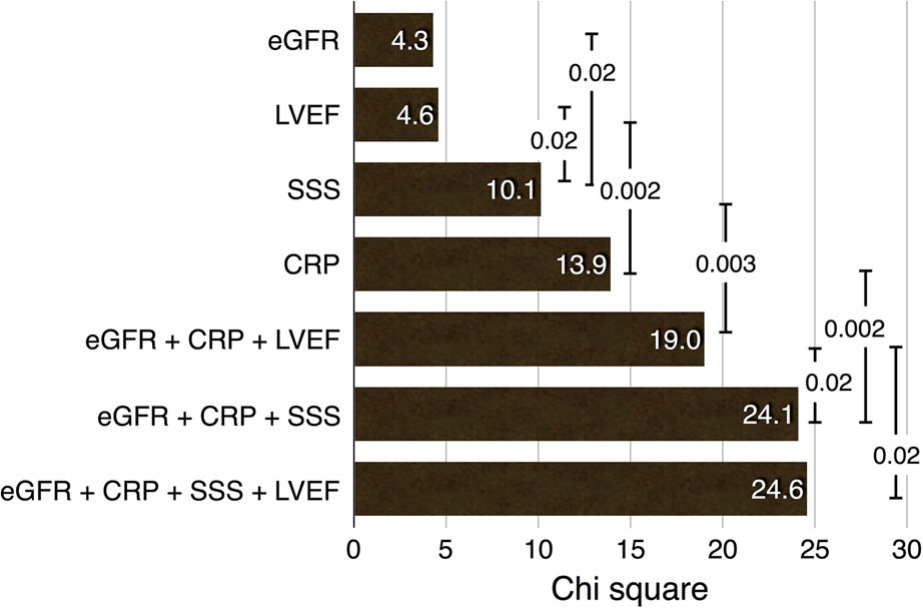
Goodness of fit of the model examined by univariate and multivariate models

**Figure 5:**
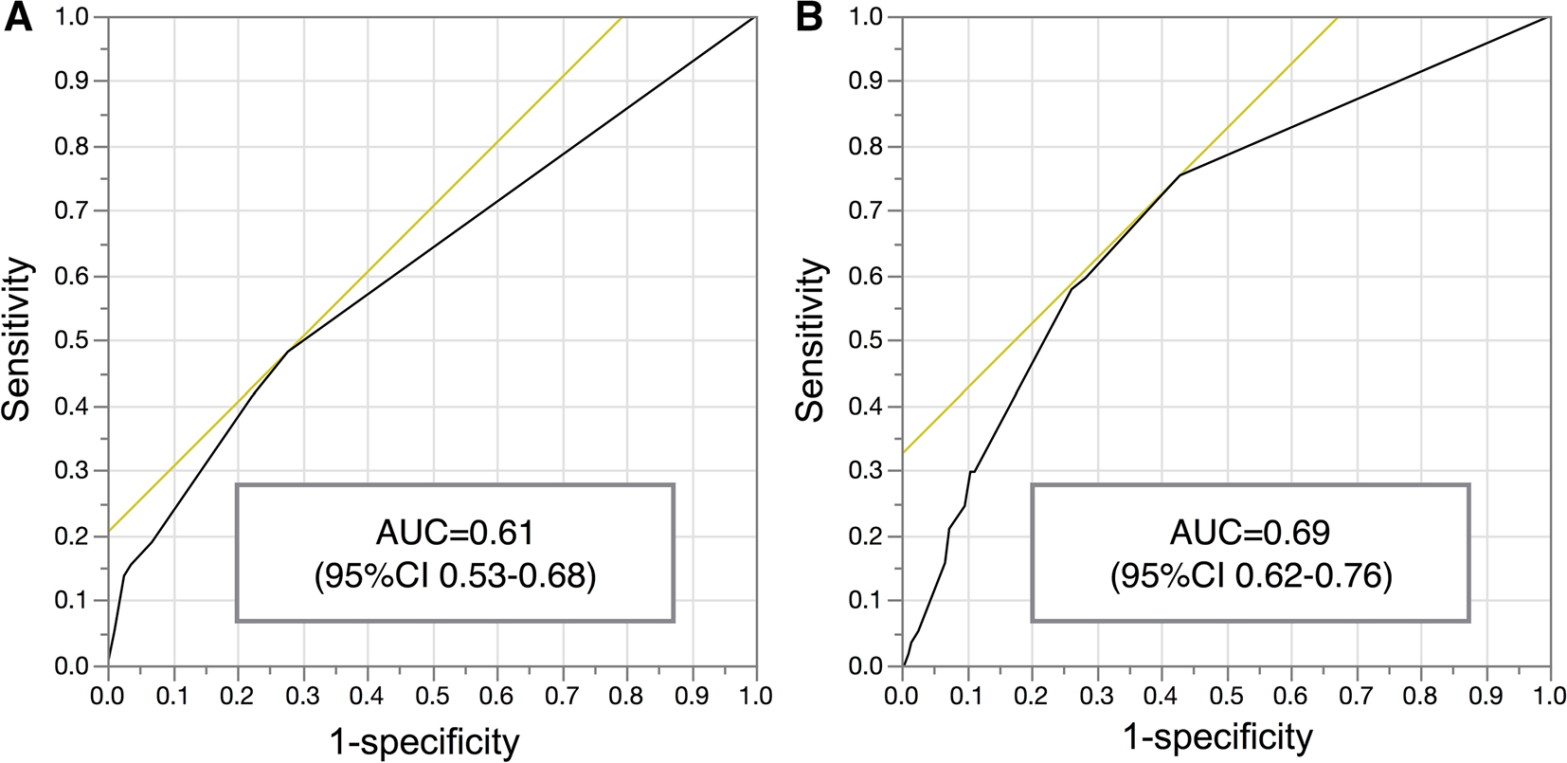
The receiver operating characteristic (ROC) analysis to predict cardiac events. **A** ROC curve of the 3-parameter model with SSS, LVEF, and eGFR. **B** ROC curve of the 4-parameter model with an additional variable of CRP. ROC AUC was significantly higher (0.61 vs 0.69, *P* < . 013) in the 4-parameter model with the addition of CRP

Goodness of fit was examined by univariate and multivariate models (Figure 4). SSS and CRP showed higher χ^2^ values compared with LVEF. When three categorical variables of eGFR, CRP, and LVEF were used, χ^2^ value was 19.0, whereas χ^2^ value was significantly increased when eGFR, CRP, and SSS were used (24.1, *P* = 0.02), showing higher incremental value of SSS compared with LVEF. In order to evaluate the effect of CRP for predicting major cardiac events, the 3-variable logistic model with SSS, LVEF, and eGFR was compared with the 4-variable model with an additional variable of CRP. The ROC AUC was significantly higher (0.61 vs 0.69, *P* < .013) in the 4-parameter model with the addition of CRP (Figure 5). Net reclassification improvement (NRI) analysis was applied to logistic models for the major cardiac events to evaluate addition of CRP. Mortality risk was divided into low and high risk (<10% and ≥10%/3 years). The majority of reclassification of adding CRP was upward reclassification of the risk, namely +24.6% (*P* < .001) for the event group (*n* *=* 57) and +11.7% (*P* < .001) for the no-event group (*n* *=* 461). The NRI of adding CRP was +12.8% and did not reach statistical significance (*P* = 0.095).

## Discussion

The present findings indicated that MPI could help predict the likelihood of cardiac events occurring during 3 years of follow-up in patients with advanced CKD, but without a definitive diagnosis of CAD. One increment of SSS represented a 9% increase in risk for cardiac events within 3 years of screening. Cardiac events increased 2.6-fold among patients with CKD and SSS ≥8 than <8. We previously showed that cardiac event risk could be estimated from MPI defect scores and LVEF in conjunction with CKD and diabetes in patients with confirmed or suspected CAD, and the increase in cardiac events was almost 2-fold higher among patients with SSS ≥9.^19^

In this study, about half of patients had cardiac symptoms or ECG abnormalities. According to the appropriate use criteria for diagnostic catheterization,^20^ these patients were considered to have intermediate or high probability of CAD. Therefore, it was appropriate to perform MPI on these patients. From the results of this study, symptoms were not helpful for predicting the cardiac events in CKD patients, but ECG changes could be a good indicator of MPI.

A clinical value of MPI has been indicated for patients undergoing hemodialysis.^4^,^12^,^21^ Indeed, a diagnosis of CAD might be problematic in the setting of CKD, since such patients frequently do not experience ischemic symptoms, elevated cardiac biomarkers, and ECG changes.^3^,^4^,^22^ Although coronary angiography has been considered the gold standard for detecting CAD, contrast media-induced nephropathy or cholesterol microembolization has emerged as serious problems.^9^ Therefore, MPI might be appropriate for CKD patients from the perspective of renal protection. Our previous studies and those of others have suggested that MPI can help predict outcomes among patients with suspected or extant CAD and those with CKD.^10^-^13^

Although the incidence and severity of CVD are increased in patients with CKD,^1^,^3^ routine screening has not yet been implemented in clinical practice. In addition, optional time-frame for screening for CVD among patients with CKD has not been established. Adjusted hazard ratios for death and cardiovascular events inversely increase with eGFR, and is especially significant in populations with eGFR <45 mL/minute/1.73 m^2.^^1^,^2^ The Suita study showed that the event rates of a first MI and stroke were about 2.5-fold higher in a Japanese urban population with eGFR <50 mL/minute/1.73 m^2^.^8^ Moreover, cardiovascular morbidity and mortality are inversely and independently associated with kidney function, particularly at eGFR <15 mL/minute/1.73 m^2^.^1^-^3^ Therefore, we selected CKD patients with eGFR <50 mL/minute/1.73 m^2^ as an appropriate target for detecting CVD, and predicted cardiac events using eGFR <15 mL/minute/1.73 m^2^.

The endpoint of the present study comprised cardiac events including cardiac death, sudden death, nonfatal MI, and hospitalization due to HF.^14^ Several studies have suggested that the incidence of CAD is >50% in unselected patients with stage 5D CKD.^3^,^23^ The risk of sudden death increases with decreasing baseline GFR.^24^ An increased prevalence of concomitant HF, ischemic heart disease, cardiac arrhythmias, and valvular calcification is associated with CKD.^3^,^25^ Because the development of HF suggests CVD in patients with CKD,^3^ the cardiac events used in this study seemed suitable as endpoints for patients with advanced CKD. Here, we used pharmacological stress MPI to evaluate patients with CKD (eGFR <50 mL/minute/1.73 m^2^) without a history of CAD, but with suspected CAD. However, the rate of hard events comprising cardiac death, sudden death, and nonfatal MI that occurred over a period of 3 years was only 2.6% (0.9%/year) when patients with severe HF were excluded. These findings indicated that the hard event rate is low for patients with CKD who do not have a history of CAD and are currently under contemporary medical therapies.

The absolute incidence and mortality rate of MI are obviously elevated in patients with advanced CKD.^3^ Standard cardiovascular risk factors are common among patients with CKD, but this cannot fully account for the high incidence of cardiovascular events and the mortality rate.^26^ One of new findings of our study is that CRP is one of important independent prognostic indicators, which has not been recognized in previous studies. Elevated inflammatory parameter such as CRP, however, could play an important role for predicting poor outcome. For example, periodontal disease, a chronic infection of the tissues surrounding teeth, contributes to the cumulated chronic systemic inflammatory burden. Recent evidence links periodontal disease with coronary heart disease and CKD.^27^ Other studies indicated that CRP predicts arterial stiffness in CKD patients.^28^ In CKD patients, the development and progression of ventricle dysfunction occurred with inflammation and mineral metabolism disorders.^29^ Inflammation and oxidative stress have been linked to the pathogenesis of plaque formation and plaque rupture,^30^ both of which are associated with worse cardiovascular outcomes.^31^ The present study found no differences in traditional risk factors between patients with and without cardiac events. Higher CRP levels were associated with cardiac events at 3 years of follow-up, but not after 1 year.^15^ These findings suggest that higher CRP levels are linked to plaque formation or plaque rupture. Hase et al. also significantly associated elevated CRP levels at the end of the predialysis phase among patients who had CKD but no CAD symptoms, with the occurrence of an initial cardiac event.^32^ Thus, inflammation might be a predictor of cardiovascular events in patients with CKD.

In conclusion, MPI can help detect CAD and predict cardiac events in CKD patients without a definitive diagnosis of CAD. Renal dysfunction combined with MPI abnormalities provides additional prognostic information that is superior to either marker alone. Higher CRP levels also predict cardiac events in patients with CKD who are not definitively diagnosed with CAD.

## Limitations

The predictive value of MPI for detecting cardiac events between patients with and without CKD could not be compared. Almost 77% of cardiac events comprised HF and volume retention due to renal failure might be involved. We could not prove that volume retention resulted in HF and not in renal failure. However, the main cause of cardiac events in Japanese patients with and without CKD was reported as severe HF.^19^ The MPI defect score was relatively lower than those previously reported.^10^^-^13 Since we excluded patients with CKD who had CAD, such a small defect might impact cardiac events in patients without such a diagnosis.

## New Knowledge Gained

All of the patients with eGFR less than 50 mL/minute/1.73 m^2^ do not need to perform MPI. Patients with eGFR <15 mL/minute/1.73 m^2^, CRP ≥0.3 mg/dL, and SSS ≥8 were significantly associated cardiac events in CKD patients with eGFR less than 50 mL/minute/1.73 m^2^. Therefore, for practical application we suggest that CKD patients with eGFR <15 mL/minute/1.73 m^2^ or CRP ≥0.3 mg/dL were indicated for MPI. If those CKD patients had SSS ≥8, their cardiac prognosis may be worse.

## Abbreviations

CAD: Coronary artery disease
CAG: Coronary angiography
CKD: Chronic kidney disease
LVEF: Left ventricular ejection fraction
eGFR: Estimated glomerular filtration rate
HF: Heart failure
MI: Myocardial infarction
MPI: Myocardial perfusion imaging
PCI: Percutaneous coronary intervention
SSS: Summed stress score
